# Oral health in Sudan: the current situation during COVID-19 pandemic

**DOI:** 10.11604/pamj.2022.41.111.32693

**Published:** 2022-02-08

**Authors:** Yasir Ahmed Mohammed Elhadi, Reem Esam Siddig, Elhadi Moheildin Awooda

**Affiliations:** 1 Department of Health Administration and Behavioral Sciences, High Institute of Public Health, Alexandria University, Alexandria, Egypt,; 2 Sudanese Medical Research Association, Khartoum, Sudan,; 3 Guarantee Educational Research and Training Centre, Gezira University, Wad Madani, Sudan

**Keywords:** COVID-19, dental care, challenges, oral health, Sudan

## Abstract

The coronavirus pandemic (COVID-19) has affected the delivery of healthcare services and posed enormous challenges to medical and dental care across the world. This article sought to explore various challenges implicated in the provision, access, and utilization of oral healthcare services in Sudan and to describe the current situation amid COVID-19. The oral health sector in Sudan has been experiencing multiple challenges in the delivery of dental care, and the current pandemic of COVID-19 has aggravated and multiplied the existing challenges. Conflict, economic meltdown, and political instability have disrupted all medical and dental services in many parts of Sudan. Furthermore, the oral health sector in Sudan suffers a lack of essential instruments, materials, and supplies, a shortage of health workers, and poor infection control practices, which present major threats to dental care in the region. The COVID-19 pandemic has contributed to further scarcity of essential materials and supplies. Moreover, the frequent closure of dental hospitals and clinics either due to civil disobedience or COVID-19 lockdown has limited accessibility and utilization of oral services. There is an urgent need to address challenges identified to ensure adequate provision of oral healthcare in Sudan.

## Commentary

The coronavirus pandemic (COVID-19) has impacted the delivery of healthcare globally, posing enormous challenges to medical and dental care across the world, and Sudan is not an exception. As of December 20, 2021, the total number of COVID-19 cases in Sudan reached 45,112 with 3,252 confirmed deaths [1]. Given the already fragile health system that was affected by years of neglect and the limited financial capacity of Sudan, COVID-19 has further impacted the delivery of essential health services [2].

According to the World Health Organization (WHO), oral health is considered a key indicator for overall health, well-being, and quality of life [3]. Following the pandemic, several mitigation strategies were proposed to curb the spread of COVID-19 and sustain emergency oral care including strict adherence to infection control practices (use of hand sanitizers, facemask, and maintaining social distancing), reducing the amount of aerosol production in dental settings, and managing the quality of air in the dental treatment rooms by reducing the use of air conditioners and improving air exchange [4].

Prompt utilization of oral services is critical for the prevention and treatment of oral diseases. However, due to the COVID-19 pandemic, dental health providers around the globe have been experiencing multiple challenges in providing oral care to their patients. Their special working conditions including proximity to patients, routine exposure to patients' airways, and performance of aerosol-generating procedures put them at increased risk of contracting COVID-19 [5]. Therefore, the delivery of dental care was significantly curtailed due to fear of contracting the disease and the closure of dental clinics [6].

Several articles have explored the status of oral care during the COVID-19 pandemic in different parts around the world, however, the situation in Sudan remains unclear. In this commentary, we aimed to highlight various challenges implicated in the provision, access, and utilization of oral healthcare services in Sudan and share the country's experience with COVID-19. This is important to identify existing challenges, summarize lessons learned and inform relevant interventions by policymakers to improve on policy and practices to ensure adequate provision of oral healthcare in Sudan.

**Overview of the oral health sector in Sudan:** dental services in Sudan are provided through public dental hospitals, dental departments in public hospitals, and private dental clinics. They are regulated by the oral health directorates under the state ministries of health in different states of Sudan. However, most of the hospitals and clinics are located in Khartoum State, the capital of Sudan. The Oral Health Directorate (OHD) is one of the Curative Medicine sectors at the Ministry of Health in Khartoum State. It is responsible for all public dental hospitals and clinics in Khartoum state. OHD has 163 public dental hospitals and outpatient clinics spread over the seven districts of Khartoum state [7].

**Challenges to the provision of oral healthcare in Sudan:** oral health sector in Sudan has been experiencing multiple challenges in the delivery of dental care summarized in Table 1, and the current pandemic of COVID-19 has aggravated and multiplied the existing challenges. It seems that the challenges to the provision of dental care due to COVID-19 are prominent in the region, posing vast threats to dental care not only in Sudan but along the entire countries of sub-Saharan Africa [8].

**Table 1:** summary of oral health challenges in Sudan and corresponding proposed solutions

Oral health challenges in Sudan	Solutions
Materials and supplies shortage	Controlling the prices of dental materials
	Encouraging the local manufacturing of dental supplies
	Facilitating the import procedures
Poor infection control practices	Raising awareness of infection control policies
	Imposing sanctions on those who do not adhere to the infection control measures
Health workforce shortage	Easing the process enrollment of graduate dentists in the internship program
	Increasing the opportunities of dental specialty training programs in the SMSB and universities
	Even distribution of the health workforce at subnational levels
Prevention and treatment of oral diseases	Oral hygiene measures
	Noninvasive treatment through fluoride application & micro-invasive fissure sealant
	Minimally invasive & Atraumatic Restorative Treatment (ART)
	Invasive use of handpiece
	Early treatment of periodontal diseases
Increased prevalence of dental trauma	Early management, education of the community about the first aid of dental trauma (especially parents and schoolteachers)
Excessive Tobacco use	Raising awareness about its´ deadly effects
	Early counseling and support for patients
	Comprehensive smoke-free policies
	Increase the prices for tobacco products

***Conflict, economic and political instability:*** the decades of war and civilian conflicts had affected the sustainable development and healthcare services in Sudan, collapsing healthcare infrastructure and poor health outcomes are some effects of the war [9]. The current transitional government in Sudan is struggling to fight against the pandemic and maintain an adequate supply of health services due to protracted conflict and protests. The civil disobedience organized by professional associations and political parties has resulted in the closure of all hospitals and clinics, leading to disruption of all medical and dental services except for the emergency [2]. The lack of control over the prices of dental materials has led to a high increase in the prices of dental services provided, particularly amid the COVID-19 pandemic.

***Materials and Supplies shortage:*** Sudan is solely dependent on imported medical and dental supplies. The economic meltdown and decreasing financial indicators hinder the production of dental materials in the country and most dental needs (instruments, materials, and other supplies) are being imported from outside Sudan. Additionally, the high inflation rate in the country has led to increased prices and a lack of technical equipment in many dental hospitals and clinics. The COVID-19 restrictions have aggravated the scarcity of dental material and resulted in the disruption of many essential oral services. Furthermore, unpaved roads throughout Sudan and floods impede movements and transportation especially through rainy reasons causing difficulties in the delivery of medicines and other essential needs for dental hospitals and clinics at peripheral States of Sudan [10, 11], the frequent power and electricity supply shortage has seriously affected the provision of dental care in Sudan.

***Health workforce shortage:*** availability of a qualified and trained health workforce is essential to the effective provision of health services. Yet before the COVID-19 pandemic, there was already a severe shortage of dental practitioners, because of the migration of qualified dental staff and recent graduates seeking overseas training. This is because a trainee in Sudan has to wait for several years after graduation to enrol in the post-graduation internship program. The main reason for this is that the number of dental colleges continues to expand, but the number of teaching hospitals and training centres available for training is not growing proportionally. Furthermore, the lack of dental materials and continuous increase in their prices is among the contributing factors, making the enrolment of graduate dentists in the internship immediately after graduation more challenging. Additionally, the scarcity of opportunities for dental speciality training, and the absence of a Sudanese dental syndicate add to the problem. More importantly, the low salaries for dental practitioners led to severe migration of qualified personnel to Arab-Gulf countries. Currently, the number of registered dentists in Sudan is 8,492 as per the latest records of Sudan Medical Council, with a 1: 33,000 dentist-to-patient ratio [12]. The issue is further complicated by the uneven distribution of the available health workforce at subnational levels; as more than one-third of the total health, the workforce is located in Khartoum, the capital city of Sudan [13].

***Poor infection control practices:*** dental procedures and instrumentation require specific strategies directed at the prevention of transmission of diseases among oral health care workers and their patients, especially during the pandemic of COVID-19. On April 18, 2020, the emergency committee in Sudan adopted precautionary preventive measures and imposed a national lockdown for 21 days. During this period all the public dental hospitals were only considering the emergency cases and private dental clinics were closed. This had resulted in a lack of accessibility to dental care services. Even after the lockdown was lifted on July 8, 2020, by the High Commission for Health Emergencies; the fear of acquiring nosocomial infection has prevented utilization of dental care by the general population due to their perceived poor infection control in dental hospitals in Sudan [14].

A recent study has reported that adherence to eye protection and the use of personal protective equipment (PPE), except gloves, is below the current recommendations for a dental practice in Sudan [15]. The vast majority of dental clinics in Khartoum state are not using high vacuum suction, the rubber dam, and the plastic barriers to cover any of the surfaces frequently touched by the dental personnel. The reasons may be lack of availability and the high cost. Due to a mandate from the Oral Health Directorate, autoclaves are widely used for sterilizing dental instruments in both public and private dental practice. However, surface disinfection is the commonly used method for sterilizing handpieces, which is far below the current recommendation for reducing the COVID-19 infection. Relying on disinfection alone is due to the time-consuming process of autoclaving and the financial costs required to buy numerous handpieces. Autoclaving of the handpiece is the preferred method, while surface disinfection and/or immersion in chemical germicides are both unacceptable methods [15]. In addition, another study reported poor quality of dental services provided in urban and rural areas of Sudan [12].

**Recommendations:** in Sudan, as is the case in most developing countries, financial constraints often lead to compromised quality of care. Therefore, health authorities should invest more in preparing the public hospitals and ensure adequate supply of all the necessary materials and instruments. Additionally, training barriers should be removed, for instance, the number of equipped dental facilities should be increased to accommodate the number of recently graduated dentists. The Medical Specialization Board should allow numerous dentists to be selected for enrolment in the speciality training programs by making use of the public centres, private and university hospitals as training centres.

The statistical report of WHO shows that until December 13, 2021, a total of 3,103,997 COVID-19 vaccine doses have been administered. Only 2% of the population were fully vaccinated due to high rates of vaccination hesitancy and other vaccination challenges in the region. It´s crucial to implement urgent measures to increase public acceptance of COVID-19 vaccines and overcome other vaccination challenges. Together with pre-existing precautionary measures, this will help to end this global pandemic and consequently mitigate the reduced access to oral healthcare services due to COVID-19.

Adherence to standards and guidelines for safe practice, and prevention of cross-infection in the dental environment, is the responsibility of all parties involved in dental treatment. The use of PPE, proper sterilization and disinfection, and following social distancing guidelines should strictly be imposed to break the chain of infection in one or more links and ensure these guidelines are following the principles of universal precautions. Tele-screening of cases and triaging should be explored as a feasible alternative, as it will help in minimizing the time of patients´ exposure to dental clinic environments, hence decreasing the risk of getting SARS-CoV-2 infection for both patients and dental healthcare workers [14].

**Conclusion:** the health system in Sudan is not equipped to handle the current circumstances of the COVID-19 pandemic. Dental public hospitals need extra supplies and instruments, moreover, there is an obvious shortage of health workers and basic protective equipment. With no compliance with preventive and infection control measures against COVID-19 infection; dental hospitals and clinics will be places for spreading the virus. Health authorities should work to improve access to health services through optimal coverage and a comprehensive and equitable geographical distribution, as well as improving the quality of health services.
